# Complete ureteral necrosis after injury sustained during lumbar disc surgery: A case report

**DOI:** 10.1097/MD.0000000000021727

**Published:** 2020-08-14

**Authors:** Jiang Fuquan, Zhang Gang, Xiao Jianlin, Ruofeng Yin

**Affiliations:** a Department of urinary surgery; b Department of Orthopaedics, China-Japan Union Hospital of Jilin University, Changchun, China.

**Keywords:** lumbar disc, ureteral injury, ureteral necrosis

## Abstract

**Introduction:**

Reports pertaining to ureteral injury sustained during lumbar disc surgery are rare; most ureteral injuries in this setting involve laceration or transection.

**Patient concerns:**

We report a rare case of a 55-year-old man who presented with complete left ureteral necrosis 20 days after sustaining ureteral transection during lumbar disc surgery.

**Diagnosis:**

The patient presented with seroperitoneum caused by left ureteral injury; post-operative histopathological examination of surgical specimen after discectomy had revealed ureter-like tissue. Exploratory laparoscopic surgery revealed necrosis of a long segment of ureter, which was not amenable to treatment with conventional methods.

**Intervention:**

: We used a spiral bladder muscle flap with vascular pedicles to repair the ureteral defect.

**Outcomes:**

Post-operative period was uneventful and the patient showed good recovery.

**Conclusion:**

Spiral bladder muscle flap with vascular pedicles may be used to repair extensive ureteric injury.

## 1 Introduction

Lumbar discectomy is an effective surgery for lumbar disc herniation syndrome. It is a relatively safe procedure with a low risk of complications such as nerve root injury, durotomy, hematoma, and wound complications.^[1,2]^ However, some rare complications of lumbo-sacral discectomy, such as ureteric injury, have been documented in some case reports.^[1,3–9]^ Most of the reported injuries involved complete or partial transection of the ureter, which can be successfully repaired with percutaneous nephrostomy or retrograde placement of ureteric stents for 6 to 8 weeks.^[3,5,8–11]^ In addition, some special types of ureteric injuries have been treated using innovative techniques such as ileal ureter replacement.^[4]^ Here, we report a rare case of ureteric necrosis resulting from injury sustained during lumbo-sacral discectomy. To the best of our knowledge, this is the first case report that documents successful use of spiral bladder muscle flap with vascular pedicles for treatment of ureteric necrosis resulting from ureteric injury sustained during lumbar discectomy.

## 2 Case report

**Figure 1:**
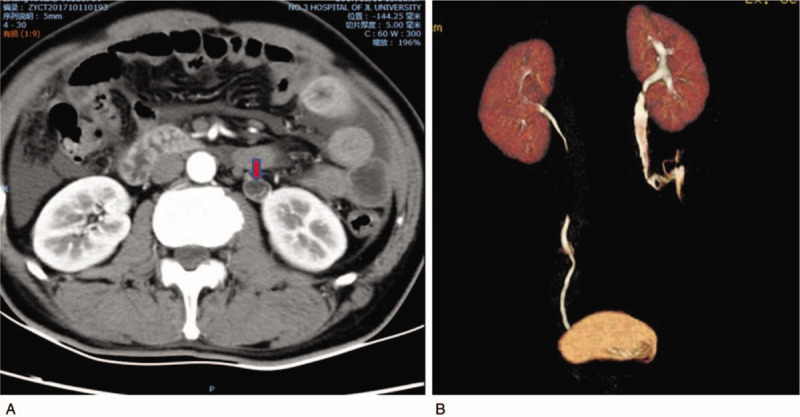
CT angiogram showing thicker proximal ureter while the distal part is not discernible; the ureter cannot be traced distally in CT. Red arrow indicates the necrotic proximal ureter with edema.

A 55-year-old man presented with seroperitoneum and left ureteral injury 20 days after undergoing Lumbar4-Lumbar5 (L4-L5) disc surgery. Histopathological examination of surgical specimen after discectomy had shown ureter like tissue. Unfortunately, no intervention was done to treat the complication until abdominal ultrasound revealed seroperitoneum and computed tomography (CT) angiography of the abdomen revealed seroperitoneum, left proximal perirenal ureter with moderate ipsilateral hydronephrosis. In addition, the proximal ureter appeared thicker while the distal ureter was not discernible; the ureter could not be traced distally (Fig. 1).

**Figure 2:**
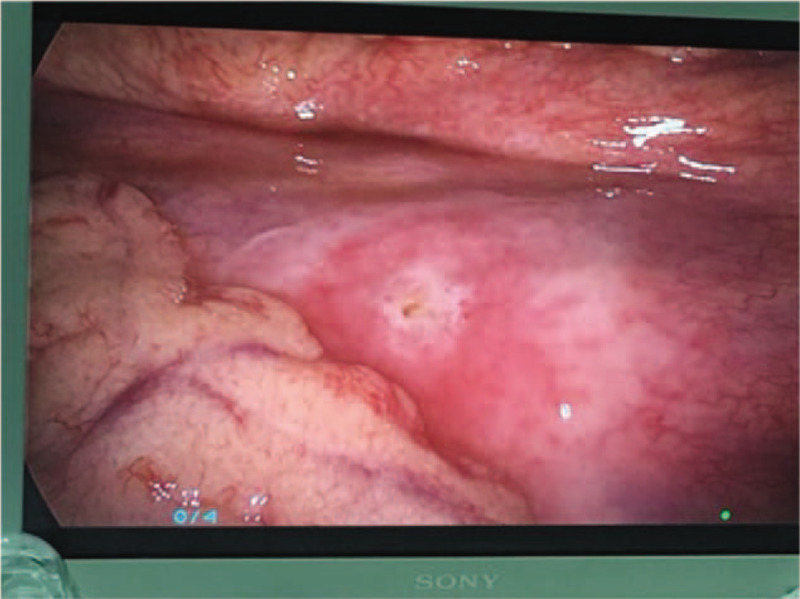
Intraoperative (laparoscopic) photograph showing the torn mesentery and presence of urine in the peritoneal cavity.

**Figure 3:**
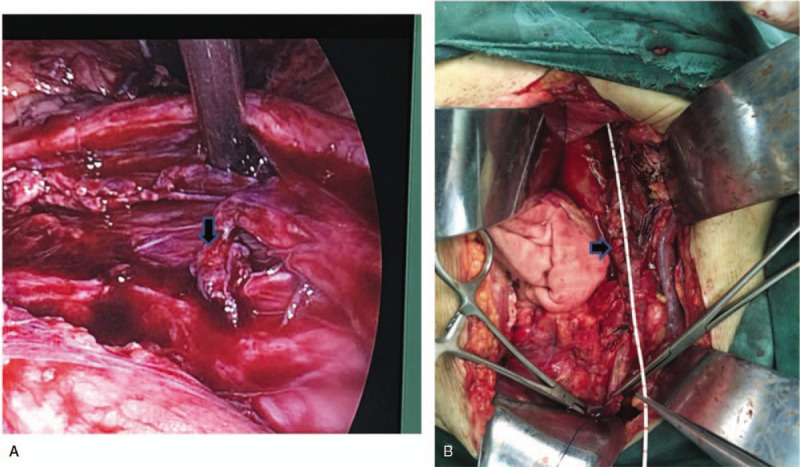
(a) Intraoperative photograph showing the remanant left distal ureter (black arrow) only 5 cm to the bladder. (b) Intraoperative photograph showing the repaired ureteral defect (black arrow); a spiral bladder muscle flap with vascular pedicles was used.

**Figure 4:**
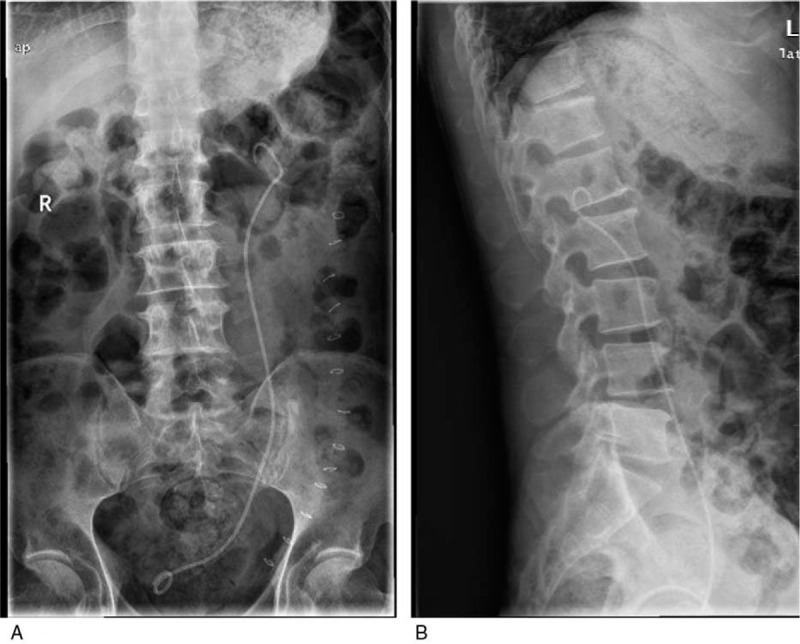
Post-operative x-ray radiograph showing the normal position of the double J stent.

**Figure 5:**
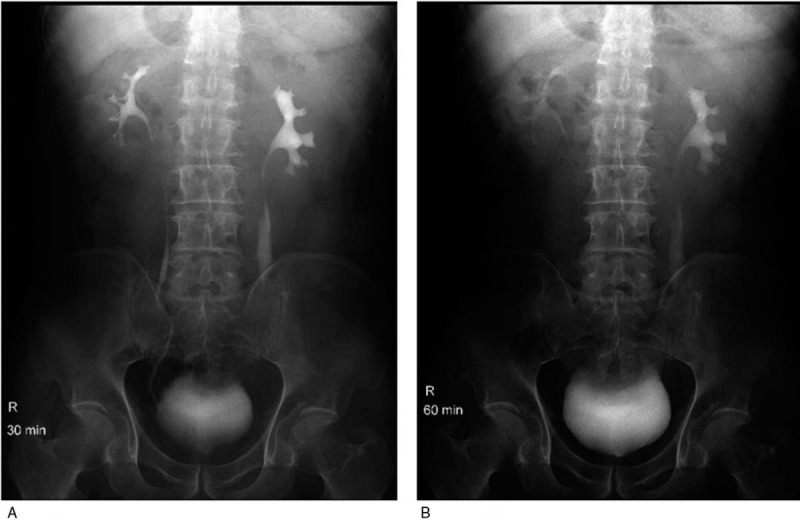
Post-operative intravenous urogram obtained at 5-month follow-up showing well-excreting left renal unit with good elimination of the contrast in postmicturation film. (a) 30 minutes after injection; (b) 60 minutes after injection.

Exploratory laparoscopic surgery revealed necrosis of a long segment of ureter, which was not amenable to treatment with conventional methods. In addition, there was presence of urine in the peritoneal cavity (Fig. 2). Exploration of the ureter through a left abdominal oblique incision revealed necrosis of a long segment of the ureter; the proximal ureteric tissue was necrotic and edematous while only the distal 5 cm of the ureter was remaining prior to its junction with the bladder. The ureter defect was not amenable to treatment using conventional methods; therefore, we used spiral bladder muscle flap with vascular pedicles along with an internal double J stent (Fig. 3). The post-operative course was uneventful. Two weeks later, post-operative anterior-posterior x-ray of the lumbar region revealed normal position of the double J stent (Fig. 4). At 5-month follow-up, intravenous urography showed excretion from the left renal unit with good flow of the contrast in postmicturation films (Fig. 5).

## 3 Discussion

This report describes a rare case of ureteric necrosis resulting from injury sustained during L4-L5 lumbar discectomy. The necrosis was successfully treated by the unusual surgery spiral bladder muscle flap with vascular pedicles, together with an internal double J stent. To the best of our knowledge, this surgical method for treatment of ureteric injury following lumbar discectomy via a posterior approach has not been reported.

The most common causes of ureteral injury during lumbar disc surgery are the following: First, anatomically, the ureter while traversing down to the bladder lays immediately anterolateral to the L4–L5 interspace. This renders the ureter susceptible to injury caused by use of pituitary rongeurs (especially those longer than 4 cm) to extract annulus fibrosus and nucleus pulposus during orthopedic surgery from the contra-lateral side. To prevent this injury, there are length marks (in centimeters) from the tip to the proximal end of pituitary rongeurs; these facilitate measurement of the distance from the posterior wall of the vertebra to the anterior longitudinal ligament. This helps ensure that the insertion length of pituitary rongeurs is less than the actual measurements to prevent any damage. Second, any defect in the anterior annulus fibrosus and/or the anterior longitudinal ligament may lower the resistance to the insertion of pituitary rongeurs due to the anterior limit of the intervertebral space. Thirdly, in patients with low body weight, the ureter rests directly on the anterior longitudinal spinal ligament between the vertebral body and the psoas muscle. Therefore, the space between the vertebra and the ureter is narrow. This is in contrast with obese patients, where the interspersed fat tissue keeps the ureter away from the intervertebral space, which helps prevent injury caused by pituitary rongeurs.^[4]^ Our patient had a thin physique, and the distance from the ureter to the posterior wall of the vertebra was 4.3 cm.

According to our literature search pertaining to ureteral injuries sustained during discectomy, the present case of ureteral necrosis is rare (Table 1). McKay first reported complete transection of ureter caused during discectomy, which was repaired with end-to-end anastmosis and stenting with good outcomes.^[5]^ A majority of the reported ureteral injuries involved lacerations or partial or complete transaction, which are amenable to treatment with end-to-end anastmosis and stenting. However, Sandoz reported a patient in whom end-to-end anastmosis and stenting failed to treat the complete transection of ureter; eventually, the patient had to undergo nephrectomy. Possible reasons for failure of end-to-end anastomosis and stenting include the following: firstly, the case was complicated with inferior vena cava injury where massive bleeding suddenly ensued from the previously lacerated lateral margin of the inferior vena cava; this may have increased the time and the difficulty of the surgery. Secondly, there was a gap of approximately 5 cm between the transected ends; thus inappropriate surgery or prolonged surgery could have resulted in failure.^[7]^ Parker and others have also reported several cases that experienced failure of treatment of complete transection of the ureter.^[9,22,23]^

**Table 1:**
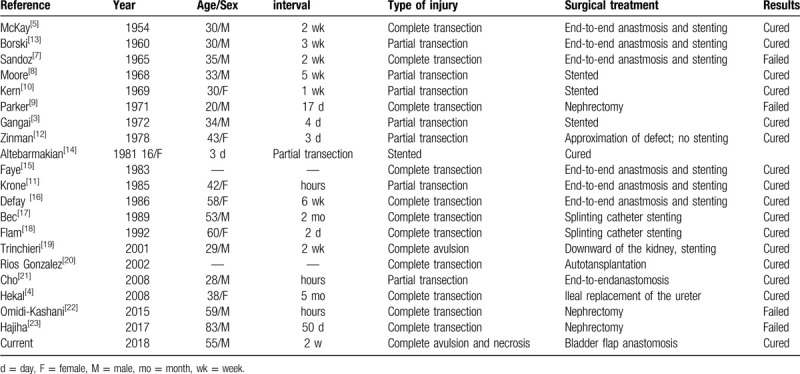
Ureteral injuries during posterior approach of disc-ectomy in the literature.

Currently, there are a few methods for surgical reconstruction of ureteral defect. Moreover, it is technically very challenging to perform autogenous kidney transplantation in the pelvic cavity due to the lack ureter and permanent nephrostomy. Normally, ileal replacement of the ureter is a commonly used method to repair ureteral defects; however, the operation is technically challenging and is associated with a high risk of complications.^[24,25]^ Zinman reported a patient who was treated with ureter approximation without stenting.^[12]^ Hekal also reported a patient with a long defect of the ureter that was successfully treated with ileal replacement.^[4]^

In our patient, we opted for spiral bladder muscle flap with vascular pedicles to treat the extensive ureteral necrosis. Owing to the vascularity of the bladder, the bladder muscle pedicle flap is a viable alternative for reconstruction of ureter. Use of autologous bladder muscle flap may facilitate regeneration of urothelial tissue. The muscle flap was designed in such a manner to ensure maximal preservation and blood flow to the upper urinary artery. This facilitated adequate perfusion of the ureter and improved postoperative outcomes. At the same time, we performed spiral suture between the bottom and top of the residual bladder wall to form a certain angle; this helped create a valve-like structure to minimize the risk of post-operative ureteral reflux. A spiral screw-shaped muscle flap was also fashioned during stitching to facilitate anterograde ureteral peristalsis in case of contraction of the bladder smooth muscle. In combination with the contractility of the helical smooth muscle suture, this helped reduce ureteral reflux, which in turn, helped prevent post-operative hydronephrosis. Finally, the patient was satisfied with the treatment outcome.

To conclude, we report the use of spiral bladder muscle flap with vascular pedicles for successful treatment of ureteral injury sustained during discectomy. Although most ureteral injuries can be treated with end-to-end anastmosis and stenting, all surgical options should be carefully considered. In case of extensive injuries, other options should be considered, such as ileal replacement of the ureter and use of spiral bladder muscle flap with vascular pedicles.

## Author contributions

**Writing - original draft:** Jiang Fuquan, Zhang Gang.

**Supervision:** Ruofeng Yin.

**Writing - review & editing:** Ruofeng Yin, Xiao Jianlin.
